# Anxiously expecting during a COVID-19: a cross-sectional descriptive inquiry on the effects of the pandemic on pregnant women

**DOI:** 10.1192/j.eurpsy.2023.978

**Published:** 2023-07-19

**Authors:** T. C. Ionescu, S. Zaharia, M. I. Draganescu, C. Tudose

**Affiliations:** 1 Department of Neuroscience, University of Medicine and Pharmacy “Carol Davila” Bucharest; 2 Department II, Alexandru Obregia Clinical Hospital of Psychiatry, Bucharest, Romania

## Abstract

**Introduction:**

While pregnancy itself is a risk factor in the development of anxiety disorders, the COVID-19 pandemic has brought additional pressure on expecting women. Despite these two independent factors, no study regarding their cumulative effect on anxiety in soon-to-be Romanian mothers exists.

**Objectives:**

This study intends to address this deficiency by measuring the level of anxiety in a sample of pregnant women from the public healthcare sector in Romania.

**Methods:**

Sociodemographic data and Zung Self-reported Anxiety Scores (SAS) were used to look at 121 pregnant women to get a fuller picture of anxiety in pregnant women during the pandemic.

**Results:**

Some of the main findings of the study are as follows: anxiety symptoms are more intense during the first trimester of pregnancy, especially in the psychological domain of the scale, as opposed to the third one. High BMI was weakly correlated with lower Zung Scale scores, while marital status and having other children were moderately correlated with less anxiety symptoms. While no association could be found between history of infection, vaccination and anxiety, surprisingly, unvaccinated women showed less psychological distress than vaccinated ones (moderate correlation), suggesting that less anxiety prone women are also less likely to get adequate protection. Getting one’s information from official sources also proved to be weakly correlated with higher Zung Scale scores.

**Image:**

**Figure:**
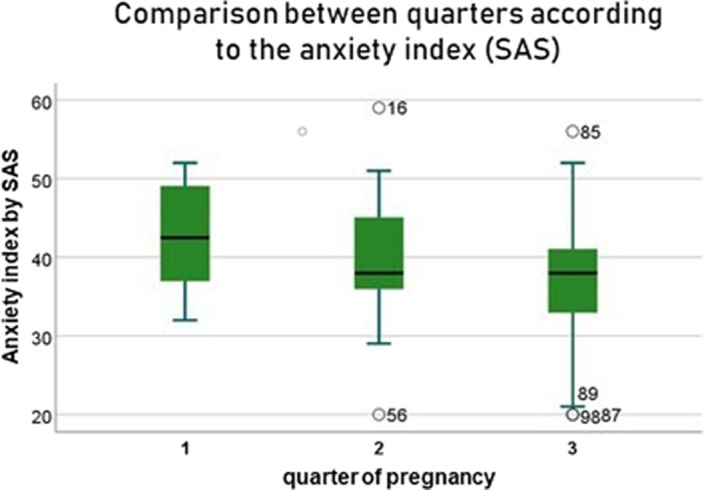

**Image 2:**

**Figure:**
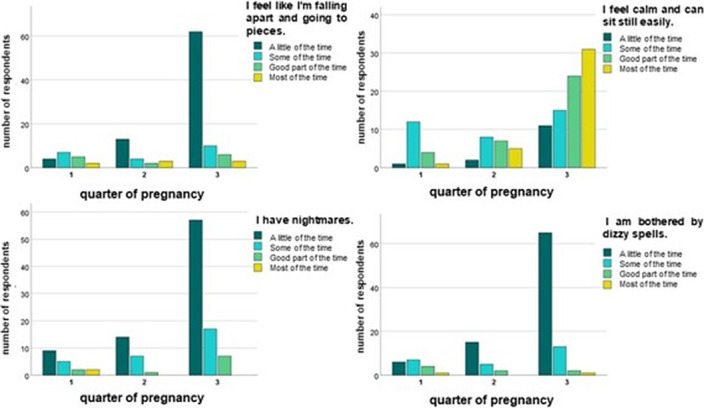

**Conclusions:**

In conclusions, the findings of this study can serve as a start for further inquiry regarding the impact of the COVID pandemic on the mental health of pregnant women.

**Disclosure of Interest:**

None Declared

